# Star-PAP regulates tumor protein D52 through modulating miR-449a/34a in breast cancer

**DOI:** 10.1242/bio.045914

**Published:** 2019-11-06

**Authors:** Aizhu Duan, Lingmei Kong, Tao An, Hongyu Zhou, Chunlei Yu, Yan Li

**Affiliations:** 1 State Key Laboratory of Phytochemistry and Plant Resources in West China, Kunming Institute of Botany, Chinese Academy of Sciences, Kunming, 650201, P.R. China; 2 University of Chinese Academy of Sciences, Beijing, 100049, P.R. China; 3 Institute of Materia Medica, School of Pharmacy, North Sichuan Medical College, Nanchong, Sichuan, 637100, P.R. China

**Keywords:** Star-PAP, TPD52, miR-449a, miR-34a, Breast cancer

## Abstract

Tumor protein D52 (TPD52) is an oncogene amplified and overexpressed in various cancers. Tumor-suppressive microRNA-449a and microRNA-34a (miR-449a/34a) were recently reported to inhibit breast cancer cell migration and invasion via targeting TPD52. However, the upstream events are not clearly defined. Star-PAP is a non-canonical poly (A) polymerase which could regulate the expression of many miRNAs and mRNAs, but its biological functions are not well elucidated. The present study aimed to explore the regulative roles of Star-PAP in miR-449a/34a and TPD52 expression in breast cancer. We observed a negative correlation between the expression of TPD52 and Star-PAP in breast cancer. Overexpression of Star-PAP inhibited TPD52 expression, while endogenous Star-PAP knockdown led to increased TPD52. Furthermore, RNA immunoprecipitation assay suggested that Star-PAP could not bind to TPD52, independent of the 3′-end processing. RNA pull-down assay showed that Star-PAP could bind to 3′region of miR-449a. In line with these results, blunted cell proliferation or cell apoptosis caused by Star-PAP was rescued by overexpression of TPD52 or downregulation of miR-449a/34a. Our findings identified that Star-PAP regulates TPD52 by modulating miR-449a/34a, which may be an important molecular mechanism underlying the tumorigenesis of breast cancer and provide a rational therapeutic target for breast cancer treatment.

## Abstract (precis)

**Summary:** Star-PAP is an important regulator of miR-449a/34a and was first identified indirectly regulating TPD52 via modulating miR-449a/34a. Furthermore, Star-PAP-miR-449a/34a-TPD52 axis is involved in proliferation and apoptosis of breast cancer cells.

## INTRODUCTION

Breast cancer is the most commonly diagnosed cancer and the leading cause of cancer death worldwide (Bray et al., 2018). Although optimization of therapy with better surgery, cytotoxic agents and endocrine drugs have highly improved the prognosis, the knowledge about the underlying mechanisms of breast cancer pathogenesis is still limited (Shiovitz and Korde, 2015; Hart et al., 2015). Therefore, a better understanding of the molecular mechanisms is imperative for discovering the potential therapeutic targets for prevention and treatment of breast cancer.

Star-PAP is a non-canonical poly (A) polymerase encoding by *TUT1*, and plays a critical role in the 3′-end processing and expression of a specific set of mRNAs and microRNAs. This process is very critical to stability and post-transcription of RNAs in human pathophysiology, including tumor progression (Li et al., 2013; Mellman et al., 2008; Knouf et al., 2013; Singh et al., 2009). Several mRNAs involved in oxidative stress response, DNA damage, apoptosis and EMT, such as HO-1, BIK and NME1 have been reported to be regulated by Star-PAP (Mellman et al., 2008; Li et al., 2017, 2012; Li and Anderson, 2014; A and Laishram, 2018). Our recent study found that Star-PAP is downregulated in breast cancer cells, and the overexpression of Star-PAP induced cell apoptosis and inhibited proliferation partly through upregulating BIK expression, suggesting that Star-PAP functions as a tumor suppressor (Yu et al., 2017). However, the other action mechanisms of Star-PAP in breast cancer still remain unknown.

In our further investigation of the regulatory mechanism of Star-PAP in tumor progression, we noticed that it was reported that Star-PAP knockdown dramatically increased Tumor protein D52 (TPD52) expression (Mellman et al., 2008). Inspired by that, TPD52 is a well-known oncogene overexpressed in breast cancer (Roslan et al., 2014; Byrne et al., 2014), we sought to determine whether regulation between Star-PAP and TPD52 was also involved in breast cancer development besides BIK. TPD52 is one member of the TPD52-like protein family, which was originally found upregulated in human breast carcinoma and further identified overexpressed in a variety of malignancies (Byrne et al., 2014; Chen et al., 2013; Tennstedt et al., 2014). Overexpressed TPD52 has been identified as a potential driver gene that is highly associated with regeneration and poor prognosis of breast cancer (Aure et al., 2013; Shehata et al., 2008a). Roslan et al. found that TPD52 was increased in breast cancer, especially in HER2 positive human breast cancer and cell lines (Roslan et al., 2014). As a kind of single-stranded non-coding RNA, microRNAs (miRNAs) play a profound role in regulating gene expression in eukaryotes. Recent studies showed that tumor-suppressive microRNA-449a and microRNA-34a (miR-449a/34a) inhibit breast cancer cell migration and invasion via targeting TPD52 (Li et al., 2016; Zhang et al., 2017). However, the upstream events that regulate these two microRNAs are not clearly defined.

Due to the important roles of Star-PAP in regulating the expression of a global miRNAs and mRNAs, the present study aimed to explore the effects of Star-PAP on miR-449a/34a and TPD52 expression in breast cancer. Our studies identified that Star-PAP regulated TPD52 through modulating miR-449/34a, which plays a critical role in inhibiting the cell proliferation and inducing the apoptosis of breast cancer cells. Our findings provided a rational therapeutic target for breast cancer treatment.

## RESULTS

### Star-PAP and TPD52 are inversely expressed in human breast cancer

**Fig. 1.**
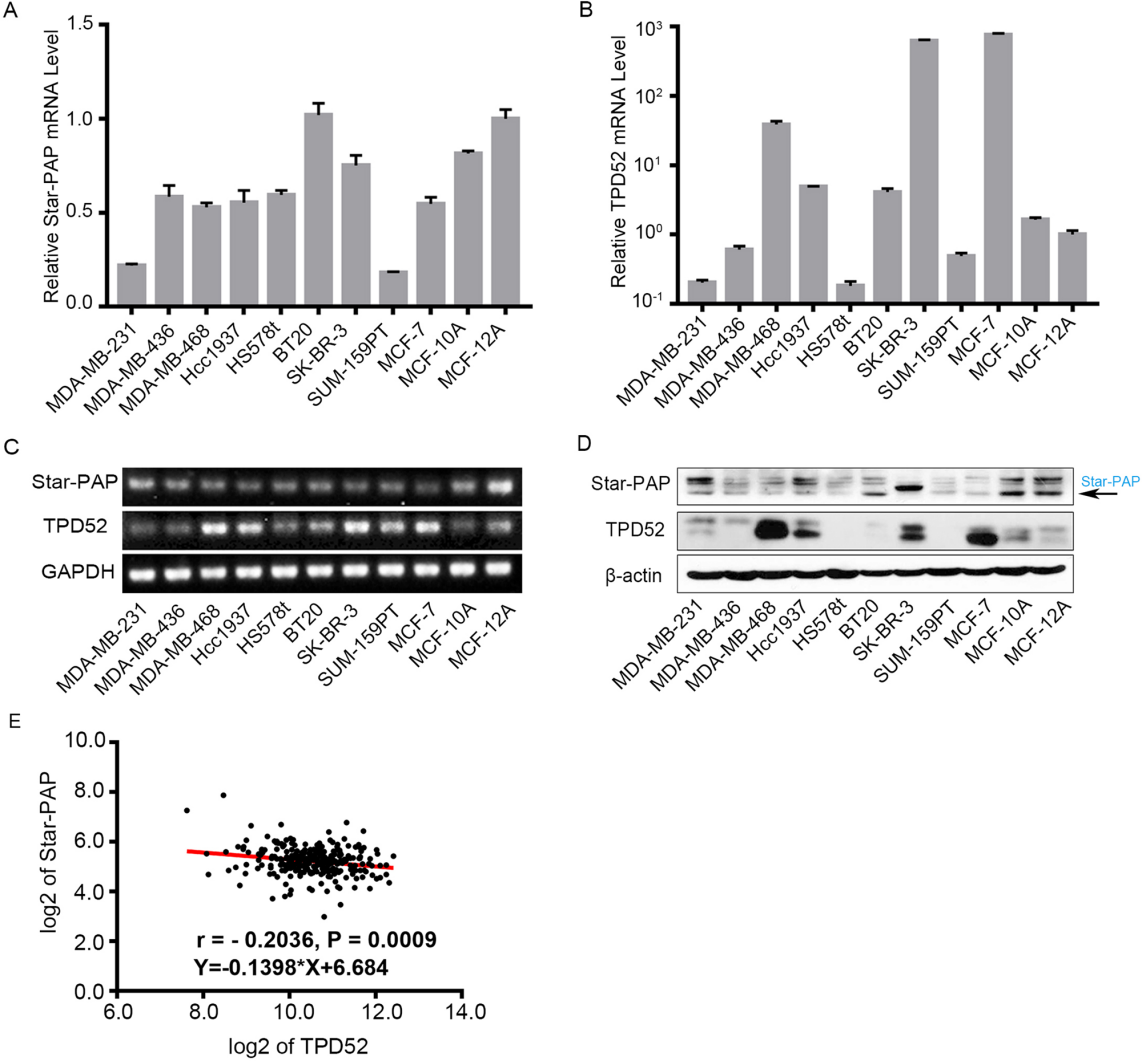
**The expressions of Star-PAP and TPD52 in breast cancer cells.** (A) Expression levels of Star-PAP mRNA in breast cancer cell lines were detected by qPCR and normalized to MCF-12A. (B) TPD52 mRNA levels were detected by qPCR and normalized to MCF-12A. (C) Star-PAP and TPD52 mRNA were examined by RT-PCR and the products were analyzed in agarose gel. (D) Protein levels of Star-PAP and TPD52 were examined by western blot. (E) The negative correlation between Star-PAP and TPD52 mRNA in 264 breast cancer patients from the R2 database.

To explore the correlation between Star-PAP and TPD52 in human breast cancer cells, we first examined the expression of Star-PAP and TPD52 in a panel of breast cancer cell lines and two mammary epithelial cell lines. Compared with the mammary epithelial cells, the breast cancer cell lines expressed lower levels of Star-PAP mRNA and higher levels of TPD52 mRNA, as quantified by qPCR (Fig. 1A,B). The results were further confirmed by the RT-PCR and western blot assays (Fig. 1C,D). Moreover, the correlation analysis of the clinical data (264 breast cancer patients) from R2 database showed that there was a negative correlation between Star-PAP and TPD52 mRNA (r=−0.2036, Y=−0.1398*X+6.684, *P*=0.0009, Fig. 1E). These data suggested that Star-PAP might play an important role in regulating TPD52 expression in breast cancer cells.

### Star-PAP regulated TPD52 expression in an indirect manner

**Fig. 2.**
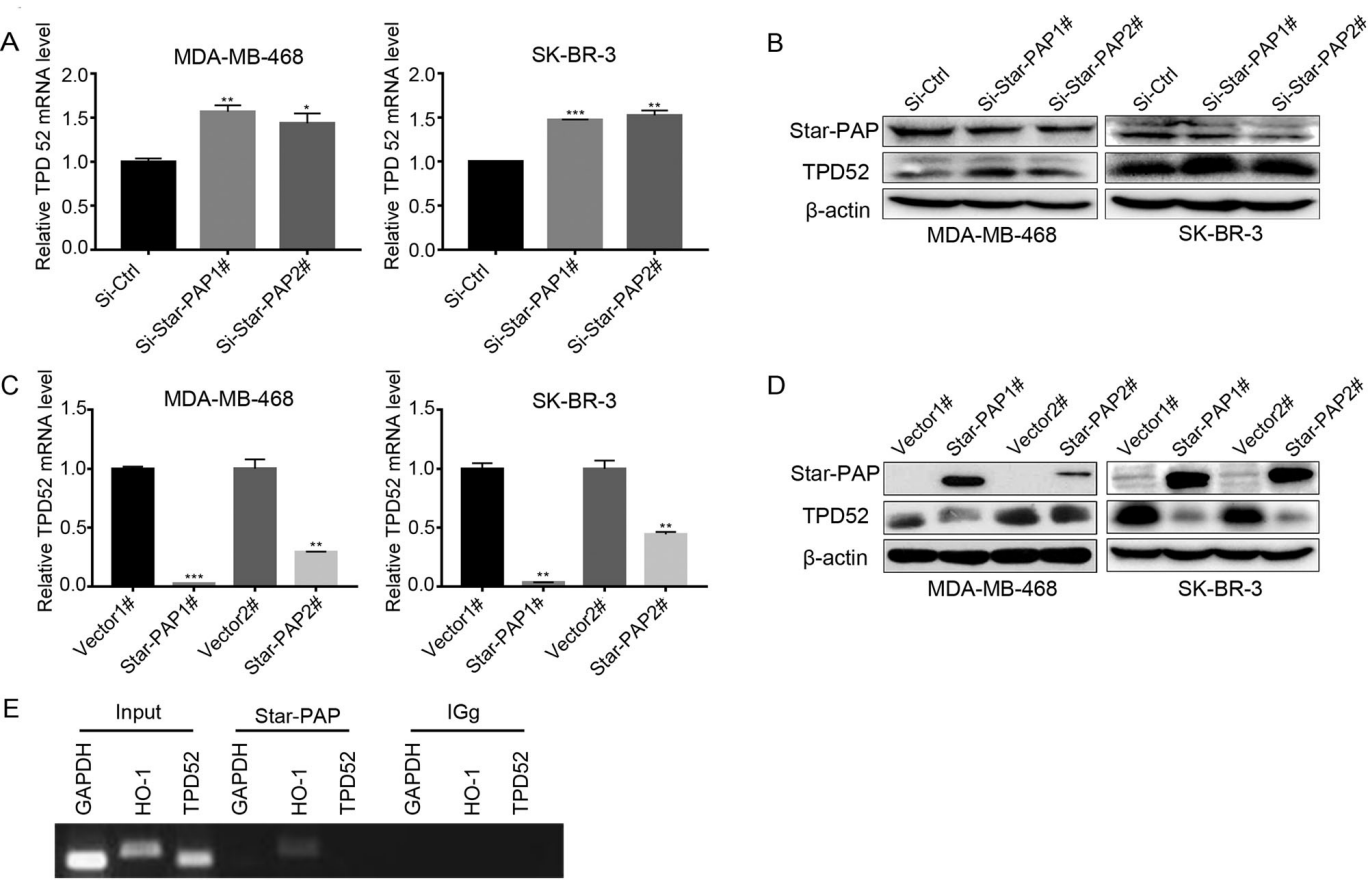
**Star-PAP regulated TPD52 expression in breast cancer cells.** (A,B) Indicated cells were transfected with Star-PAP siRNAs for 48 h, the TPD52 mRNA level (A) and protein level (B) were detected by qPCR and western blot. (C,D) Cells were transfected with plasmids inserting with *Star-PAP* for 24 h, then TPD52 mRNA (C) and protein level (D) were detected by qPCR and western blot. (E) TPD52 mRNA was detected by RT-PCR after RIP of Star-PAP in SK-BR-3 cells. GAPDH (non-Star-PAP target mRNA) and HO-1 (Star-PAP target mRNA) were used as the negative and positive control, respectively. Data are means±s.d. (*n*=3) with three independent repeats, *P<0.05, **P<0.01, ***P<0.001.

To elucidate the regulatory effect of Star-PAP on TPD52, siRNA targeting *Star-PAP* was employed to knockdown the endogenous Star-PAP in MDA-MB-468 and SK-BR-3 cells. As the result, downregulation of Star-PAP led to increased expression of TPD52 in both cell lines (Fig. 2A,B). In contrast, when Star-PAP was overexpressed, the expression of TPD52 mRNA and protein levels were both markedly reduced (Fig. 2C,D). The above-mentioned results suggested that Star-PAP is an important regulator of TPD52. Given the role of Star-PAP in processing the 3′-end of some specific mRNAs, we employed RNA immunoprecipitation (RIP) assay to investigate whether Star-PAP directly bind to TPD52 mRNA. Unexpectedly, the data showed that Star-PAP could bind to its direct target gene HO-1 but could not bind to TPD52, suggesting that Star-PAP may regulate TPD52 expression in an indirect manner (Fig. 2E).

### TPD52 was modulated by miR-449a and miR-34a

**Fig. 3.**
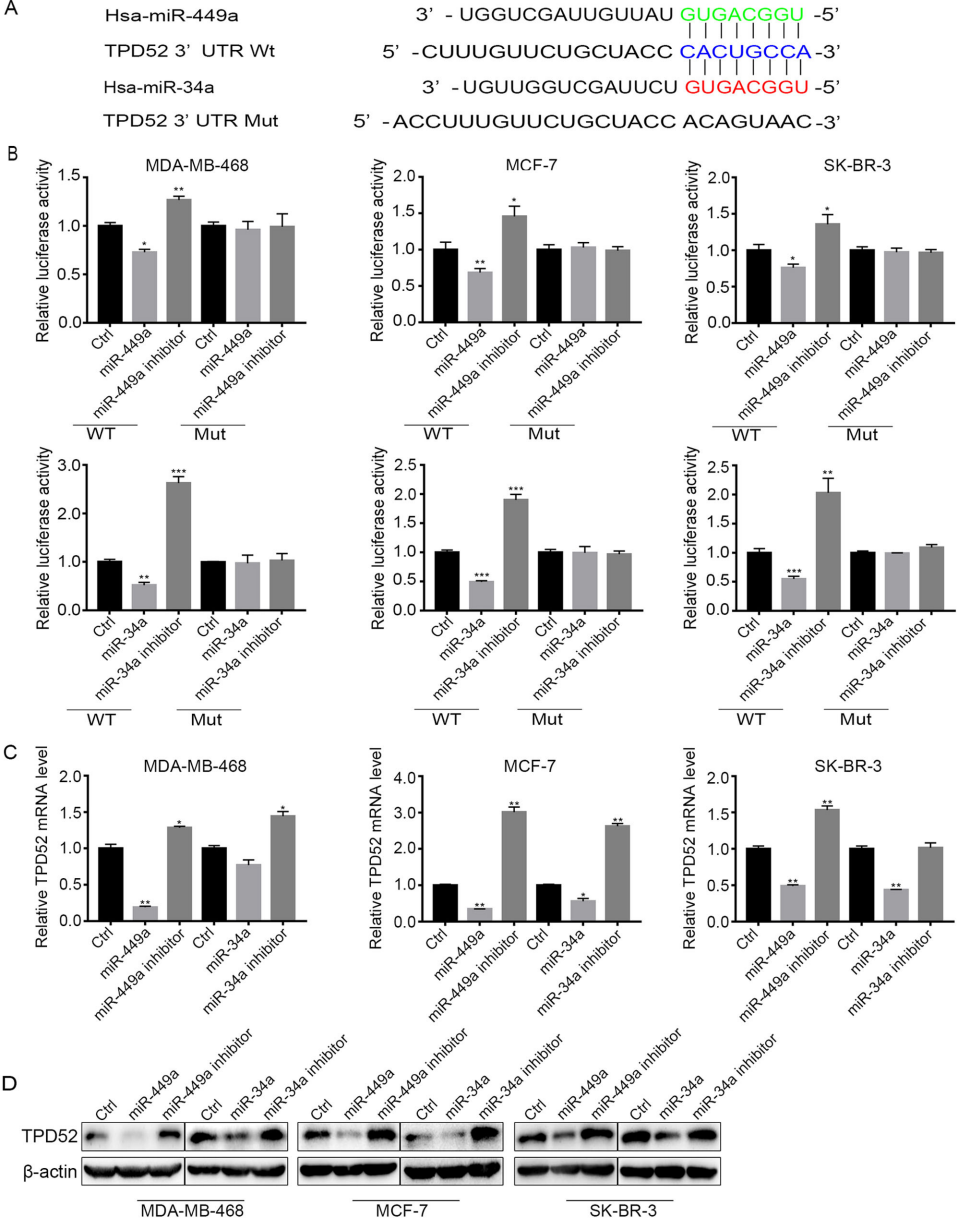
**MiR-449a/34a inhibited TPD52 expression.** (A) The predicted miR-449a/34a binding sequences and the mutant sequence at the 3′-UTR of TPD52 mRNA. (B) Cells were co-transfected with either 50 nM miR-449a/34a mimics or inhibitors and 80 ng plasmid carrying either WT or Mut 3′-UTR of TPD52. The relative firefly luciferase activity normalized with Renilla luciferase was measured 48 h after transfection. (C,D) Cells were transfected with miR-449a/34a mimics or inhibitors for 48 h, and the expressions of TPD52 were detected by qPCR (C), and the protein levels were detected by western blot (D). Data are means±s.d. (*n*=3) with three independent repeats, *P<0.05, **P<0.01, ***P<0.001.

To clarify the regulatory mechanism of TPD52 in breast cancer, Targetscan (http://www.targetscan.org/ V7.0) was used to predict the regulator(s) of TPD52. Bioinformatics prediction showed that there was a putative sequence region in miR-449a and miR-34a to bind 3′-untranslated region (UTR) of TPD52 (Fig. 3A). To confirm the binding, we constructed firefly luciferase reporters containing the wild type or 3′-UTR-mutant of TPD52. The dual luciferase reporter assays were performed in MDA-MB-468, MCF-7 and SK-BR-3 cell lines. In all the tested cell lines, when the wild-type TPD52 was co-transfected with miR-449a/34a mimics could significantly inhibit the relative luciferase activity, while miR-449a/34a inhibitors increased luciferase activity (Fig. 3B). Whereas, mutation of the perfectly complementary sites in the 3′-UTR of TPD52 abolished the suppressive or upregulated effects of miR-449a/34a mimics and inhibitors, respectively (Fig. 3B). These results indicated that miR-449a/34a could suppress the expression of TPD52 by interacting with its 3′-UTR complementary sequences. Moreover, in the same cell lines, the transfection of miR-449a/34a mimics or inhibitors could decrease or increase the expression levels of TPD52 mRNA and protein, respectively (Fig. 3C,D). Taken together, these data suggested that TPD52 was directly modulated by miR-449a and miR-34a in human breast cancer cell lines.

### Star-PAP regulated TPD52 by modulating miR-449a/34a

**Fig. 4.**
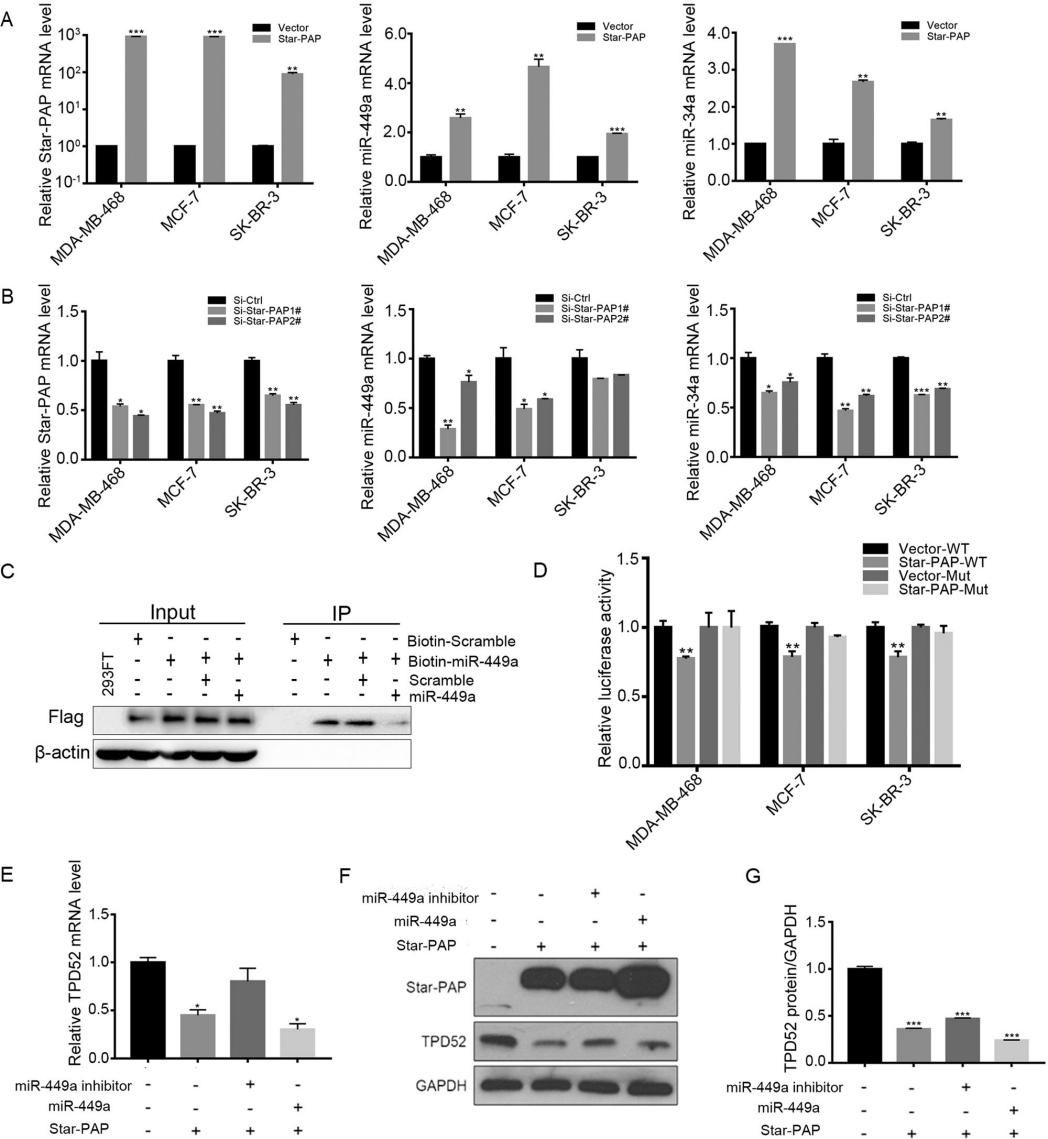
**Star-PAP regulated miR-449a/34a expressions.** (A) Overexpression of Star-PAP for 24 h. The expression of miR-449a/34a was detected by qPCR. (B) Decreased levels of miR-449a/34a following Star-PAP knockdown by siRNAs for 48 h. (C) Cells were co-transfected with either pFlagcmv2-Star-PAP and 80 ng plasmid carrying either WT or Mut 3′-UTR of TPD52. The relative firefly luciferase activity normalized with Renilla luciferase was measured 24 h after transfection. (D) RNA pull down assay was performed by 5′-biotin-miR-449a and biotin-Scramble. 293 FT cells were transfected with pFlagcmv2-Star-PAP for 24 h. Cells were lysed in RIPA buffer, then incubated with biotin-miR-449a or biotin-scramble for 4 h, before that they were pre-incubated with miR-449a or Scramble for 1 h, followed by adding avidin beads, and finally examined by western blot. (E) Cells were co-transfected with Star-PAP and miR-449a mimic or inhibitor, the TPD52 mRNA level was detected by qPCR. (F) The same treatment as with E, and the protein levels were examined. (G) Quantification of TPD52 protein level in F by NIH ImageJ software. Data are means±s.d. (*n*=3) with three independent repeats, *P<0.05, **P<0.01, ***P<0.001.

A previous study showed that the suppression of Star-PAP resulted in an approximately 40% decrease in the global miRNAs levels, as measured by real-time PCR-based miRNA profiling (Knouf et al., 2013). In this work, we found that when Star-PAP overexpressed in the three cell lines, the levels of both miR-449a and miR-34a were significantly upregulated (*P*<0.01 versus control) (Fig. 4A). Moreover, the expression levels of miR-449a/34a were both significantly downregulated following Star-PAP knockdown (Fig. 4B). To further clarify the underlying mechanisms, RNA pull-down assay was performed and the result showed that Star-PAP could selectively bind to 3′region of miR-449a (Fig. 4C). Accordingly, we suggested that Star-PAP may regulate TPD52 through modulating miR-449a/34a. Subsequently, the dual luciferase reporter assay was conducted, and the results showed that the co-transfection of Star-PAP with wild-type TPD52 reduced the relative luciferase activity (Fig. 4D). Whereas, co-transfection of Star-PAP with TPD52 3′-UTR mutation had no effects on the luciferase activity (Fig. 4D). Moreover, the decreased levels of TPD52 mRNA and protein caused by Star-PAP were rescued or aggravated by the co-transfection with miR-449a inhibitor or miR-449a mimic, respectively (Fig. 4E,F). Taken together, the results indicated that Star-PAP suppressed the expression of TPD52 by a miR-449a/34a-mediated mechanism.

### Star-PAP suppressed breast cancer cell growth and promoted apoptosis through inhibiting TPD52 expression

**Fig. 5.**
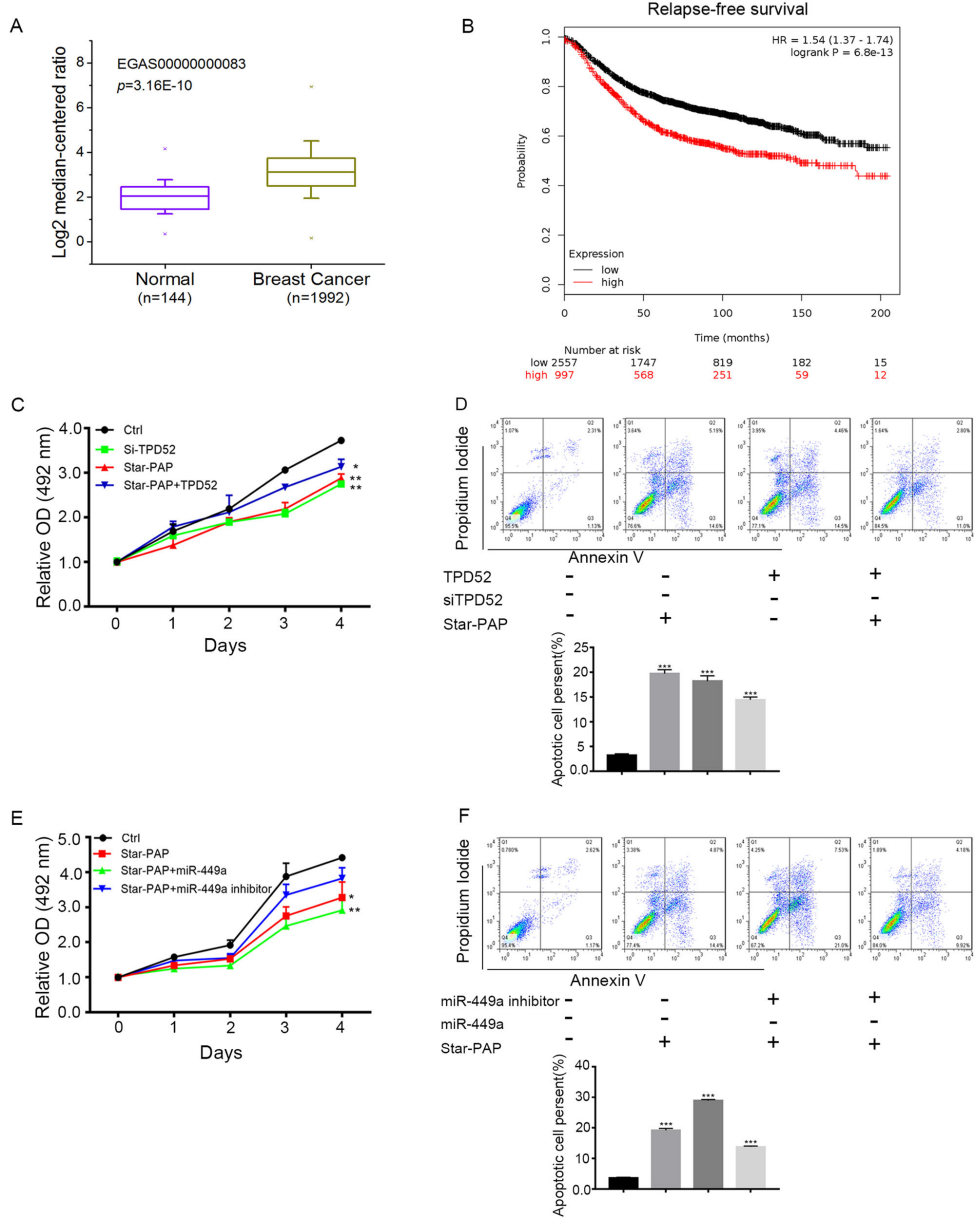
**Star-PAP suppressed MDA-MB-468 proliferation and induced apoptosis through inhibiting TPD52 expression.** (A) Expression levels of TPD52 in 1992 breast cancer patients and 144 healthy donors. Dataset accession number and *P*-value were shown. (B) KM-plotter analysis of relapse-free survival of breast cancer patients stratified by the expression levels of TPD52. Number of patients, log-rank *P*-value and hazard ratio (HR) were shown. (C) Cells transfected with Star-PAP, siTPD52 or co-transfected with Star-PAP and TPD52 were seeded in 4000/well, and cell viability was measured by MTS. (D) The same treatment with C for 48 h, and the apoptotic cells were analyzed by Flow cytometry. (E) Cells transfected with Star-PAP and miR-449a mimic or inhibitor were seeded in 4000/well, and cell viability was measured by MTS. (F) The same treatment as C for 48 h, and cell apoptosis was analyzed. Data are means±s.d. (*n*=3) with three independent repeats, *P<0.05, **P<0.01, ***P<0.001.

As a potential driver gene, TPD52 is highly associated with regeneration in breast cancer (Aure et al., 2013). In order to investigate the correlation between TPD52 levels and clinical prognosis, the expression pattern of TPD52 was firstly analyzed in Oncomine database. The breast cancer patients (*n*=1992) expressed significantly higher levels of TPD52 than the normal donors (*n*=144) (Fig. 5A). Then KM-plotter, an online cancer survival analysis database, was further exploited to evaluate the relationship between TPD52 expression and the prognosis across the entire spectrum of breast cancer patients, and the results showed that high levels of TPD52 was associated with relatively decreased relapse-free survival (Fig. 5B). In addition, the elevated TPD52 expression also indicated a lower relapse-free survival in the four major breast cancer subtypes (basal-like, HER2-enriched, luminal A and luminal B) (Fig. S2). We also found that TPD52 expression was significantly higher both in NSCLC and ovarian cancer. Consistently, the poor prognosis was also observed in TPD52-high NSCLC or ovarian cancer patients (Fig. S3). Collectively, these data indicated that the upregulation of TPD52 was positively correlated with the poor prognosis in various types of cancer.

Considering the regulatory relationship between Star-PAP and TPD52, we hypothesized that the tumor suppressing effect of Star-PAP in breast cancer cells might be mediated by modulating TPD52 expression. Indeed, Star-PAP overexpression suppressed cell proliferation and promoted apoptosis, which was similar to the effects of TPD52 downregulation (Roslan et al., 2014; Han et al., 2015; Chen et al., 2016). Whereas the inhibitory effect of Star-PAP overexpression was reversed by co-transfection with TPD52 (Fig. 5C,D). Furthermore, to confirm the roles of miR-449a/34a in the Star-PAP-TPD52 pathway, Star-PAP was co-transfected with miR-449a mimic or inhibitor. The miR-449a mimic exhibited synergistic effects with Star-PAP on cell proliferation suppression and apoptosis promotion. In contrast, the effects of Star-PAP were weakened by the co-transfection of miR-449a inhibitor (Fig. 5E,F). Similar results were obtained when the cells were co-transfected with miR-34a (Fig. S1). These data verified that Star-PAP regulated TPD52 via modulating miR-449a/34a, as a molecular mechanism underlying the function of Star-PAP as a tumor suppressor gene.

## DISCUSSION

For the first time, we illuminated the regulatory relationship among Star-PAP, miR-449a/34a and TPD52. We found that Star-PAP indirectly regulated TPD52 through modulating miR-449a/34a. TPD52 is an oncogene overexpressed in various cancers, including breast, prostate, lung, gastric, ovarian and pediatric cancers (Byrne et al., 2014; Shehata et al., 2008b; Chen et al., 1997; Cheng et al., 2012). Knockdown of TPD52 induced G1 arrest or disrupted the mitochondrial membrane potential and activated caspase 9 and caspase 3 (Chen et al., 2016; Ummanni et al., 2008). In the present study, we found that TPD52 is generally upregulated in breast cancer cell lines, especially in MDA-MB-468, MCF-7 and SK-BR-3 cells (Fig. 1B). Consistently, we also found that TPD52 was significantly upregulated in breast cancer samples (Fig. 5A).

Recently, miRNAs were reported to suppress the expression of TPD52 via its 3′-UTR at transcriptional level (Byrne et al., 1995). MiR-449a/34a have been reported to inhibit breast cancer cell migration and invasion through targeting TPD52 (Li et al., 2016; Zhang et al., 2017). Since miR-449a and miR-34a share the same seed sequence, they are expected to regulate the overlapping target genes (Bou Kheir et al., 2011). Herein, we found that miR-449a and miR-34a could inhibit cell proliferation of breast cancer cells through suppressing TPD52. The expression level of TPD52 was decreased by transfecting with miR-449a/34a mimics but increased with miR-449a/34a inhibitors (Fig. 3B–D). MiR-34a and miR-449a played key roles in tumorigenesis, specifically, miR-34a has been described as a master regulator of tumor suppression (Bader, 2012). Although the bioactivity of these miRNAs has been well studied, their upstream regulatory events are not clearly defined.

Modification of miRNA sequences by the 3′ addition of nucleotides is a conserved physiological and common post-transcriptional event that shows selectivity for specific miRNAs and is also observed across species ranging from *Caenorhabditis elegans* to human (Wyman et al., 2011). The modifications resulting predominantly from adenylation and uridylation could influence miRNA stability and efficiency of target repression (Wyman et al., 2011). As a poly (A) and poly (U) polymerase, Star-PAP regulates both 3′A and 3′U additions in a variety of substrates, including miRNAs and mRNAs (Mellman et al., 2008; Trippe et al., 1998; Wyman et al., 2011). For example, the suppression of Star-PAP could significantly reduce miR-200a with a 3′U and miR-31 with 3′A in HCT-116 cells (Wyman et al., 2011). A previous study suggested that Star-PAP suppression resulted in a global decrease in miRNA levels of approximately 40%, as measured by real-time PCR-based miRNA profiling (Knouf et al., 2013). Herein, we evaluated the effects of Star-PAP on miR-449a and miR-34a in breast cancer cells, and the results showed that both miR-449a and miR-34a were upregulated following Star-PAP overexpression, while they were reduced by Star-PAP knockdown (Fig. 4A,B). It has been found that Star-PAP modifies miRNAs 3′ nucleotide additions and as opposed to the canonical poly (A) polymerases (PAPs), Star-PAP directly binds its target and plays a structural role to help assemble the cleavage and polyadenylation complex (Wyman et al., 2011; Laishram and Anderson, 2010). Consistently, we found that Star-PAP directly binds to the 3′ region of miR-449a, as identified by RNA pull-down assay (Fig. 4C). Our results proved that Star-PAP is an important regulator of miR-449a and miR-34a in human breast cells.

mRNA maturation involves the addition of a poly (A) tail (∼250 adeno sine residue) at the 3′-end by PAPs in the nucleus (Zhao et al., 1999; Edmonds, 2002; Laishram, 2014; Moore and Proudfoot, 2009; Proudfoot and O'Sullivan, 2002), which is essential for mRNA stability, translation and exporting the transcript from the nucleus to the cytoplasm (Zhao et al., 1999; Laishram, 2014; Proudfoot, 2004). Previous studies have shown that mRNA maturation of several apoptosis-related genes, such as BIK, was regulated by Star-PAP. Our previous work showed that Star-PAP controls BIK expression and induced cell apoptosis through the mitochondrial pathway (Yu et al., 2017). However, investigation of the biological functions of mRNA 3′-end processing by Star-PAP is still at a nascent stage. Given that TPD52 is closely associated with tumor progression, our study here identified a clearly regulatory relationship that Star-PAP regulates the expression of TPD52 via modulating miR-449a/34a, rather than the direct 3′-end processing way in breast cancer cells (Fig. 2E). Furthermore, the proposed Star-PAP-miR-449a/34a-TPD52 axis is involved in proliferation and apoptosis of breast cancer cells (Fig. 5; Fig. S1). Our findings thus provide a potential therapeutic target for breast cancer treatment.

## MATERIALS AND METHODS

### Cell lines and cell culture

All breast cancer cell lines except SUM-159PT were obtained from ATCC (Manassas, VA, USA) and authenticated by short tandem repeat profiling. MDA-MB-231, MDA-MB-436, MDA-MB-468, BT-20 and MCF-7 were cultured in DMEM supplemented with 10% FBS and 1% penicillin/streptomycin (P/S) (HyClone, Logan, UT, USA). HCC1937 and SK-BR-3 were cultured in RPMI 1640 with 10% FBS and 1% P/S. MCF-10A and MCF-12A were cultured in DMEM/F12 supplemented with 5% horse serum, 20 ng/ml EGF, 100 ng/ml cholera toxin, 10 μg/ml insulin, 500 ng/ml hydrocortisone. SUM-159PT was obtained from Asterand (Hertfordshire, UK) and cultured in Ham's F12 medium supplemented with 5% FBS, 1 μg/ml hydrocortisone and 5 μg/ml insulin. HS578t was cultured in DMEM with 10% FBS, 10 μg/ml insulin, and 30 ng/ml EGF. All cell lines were mycoplasma free and passaged no more than ten passages after resuscitation.

### Plasmids, siRNAs and luciferase reporter assay

Lipofectamine 3000 (Thermo Fisher Scientific, Waltham, MA, USA) was used for both siRNA and plasmid transfection according to the manufacturer's instructions. Star-PAP cDNA (GeneCopoeia, Rockville, MD, USA) was cloned into pFlag-CMV2 and pCDNA6-myc-his vector for transient overexpression. Two reported siStar-PAPs were used for knockdown, and the sequences were as follows: siStar-PAP-1: 5′-AACUACGAGCTGCGAGAAA-3′; siStar-PAP-2:5′-GUGUGUUUGUCAGUGGCUU-3′; and siTPD52: 5′-GGAAGAGCUAAGAGAATT-3′. Luciferase reporter assays were done in 96-well plates with three repeats. Cells treated with control, miR-34a/449a mimics, or miR-34a/449a inhibitors (GenePharma, Shanghai, China), or Star-PAP pFlag-CMV2 were co-transfected with wild type (TPD52 3′UTR Lenti-reporter-Luc Vector, abm) or mutant of TPD52 3′-UTR luciferase reporters together with Renilla plasmid. Cells were lysed after transfection and the relative firefly luciferase activity normalized with Renilla luciferase by using the Dual-Luciferase Reporter Assay kit (Promega, Madison, WI, USA).

### qPCR and RT-PCR assay

Total RNA was prepared with TRIzol (Thermo Fisher Scientific) according to the manufacturer's protocol. Reverse transcription was performed using RevertAid H Minus First-Strand cDNA Synthesis Kit (Thermo Fisher Scientific). For qPCR, SYBR Select Master Mix (Roche) was used with ABI 7500 Real-Time PCR System (Thermo Fisher Scientific). For RT-PCR, PrimeSTAR HS DNA Polymerase (Takara, Kusatsu, Japan) was used, and the products were analyzed in agarose gel. For detection of mature miR-449a/34a, total RNA was subjected to reverse TaqMan MicroRNA Reverse Transcription Kit (Applied Biosystems), and TaqMan MicroRNA Assay Kit (Applied Biosystems) was used for qPCR analysis. U6 or GAPDH was measured as an internal control for miRNA or mRNA. Relative changes in gene and miRNA expression were determined using the 2^−ΔΔ^Ct method. The primers for qPCR and RT-PCR analysis of mRNA were:

GAPDH-F: 5′-ACGACCACTTTGTCAAGCTCA-3′, GAPDH-R: 5′- TCTCTCTTCCTCTTGTGCTCT-3′; Star-PAP-F: 5′-GAGTTCTTCCCTGGCTGTGT-3′, Star-PAP-R: 5′- AGCGATGGAGATTCTGGAGC-3′; TPD52-F: 5′- ACTACCAGTCCCCGTTTGATT-3′, TPD52-R: 5′-CTCAGGGACTGGGTCTGTTC-3′; HO-1-F: 5′-CCACCAAGTTCAAGCAGCTCTA-3′, HO-1-R: 5′-GCTCCTGCAACTCCTCAAAGAG-3′.

### RNA immunoprecipitation assay

RIP was performed as reported with antibody against Star-PAP (Yu et al., 2017). A total of 10^7^ cells were used for the preparation of mRNP lysate. Finally, RNA was purified with TRIzol (Thermo Fisher Scientific) and reverse transcribed by RevertAid H Minus First Strand cDNA Synthesis Kit (Thermo Fisher Scientific).

### Western blotting assay

Cells were seeded in six-well plates with a density of 4-5×10^5^cells/well and transfected with indicated plasmids or microRNAs for 24–48 h. Cells were lysed in RIPA buffer (50 mM Tris-HCl [pH 7.4], 150 mM NaCl, 1% sodium deoxycholate, 0.1% sodium dodecyl sulfate, 1% NP-40, 1 mM EDTA and 1 mM PMSF), which contained protease inhibitor (Roche) and protein concentration was determined by BCA^TM^ protein assay kit (Thermo Fisher Scientific). Western blotting was performed with Primary antibodies for Star-PAP and TPD52 (Abcam, Cambridge, UK), β-actin and GAPDH (Sigma-Aldrich, St Louis, MO, USA) were used according to the manufacturer's instructions.

### RNA pull-down assay

HEK-293 FT cells were seeded in a 6 cm dish, and transfected with Star-PAP for 24 h. Cells were then lysed in RIPA buffer (0.01 M Tris, 0.04 M NaCl, 0.005 M EDTA, Triton X-100) containing protease inhibitor and PMSF. Cell supernatants were then incubated with 5′ biotin-449a or biotin control (GenePharma) in 4°C for 4 h, followed by adding avidin beads (Thermo Fisher Scientific) overnight. Finally, the beads were washed and boiled for western blot detection.

### Cell proliferation assay

MDA-MB-468 cells were trypsinized and reseeded into 96-well plates at the concentration of 4000 cells/well in triplicate after transfection. Absorbance at 490 nm was firstly measured using MTS (Promega, Madison, WI, USA) 4 h later, and then measurements were taken every 24 h.

### Cell apoptosis analysis

Cell apoptosis was analyzed by the Annexin V-FITC/Propidium Iodide (PI) Apoptosis kit (BD Biosciences, Franklin Lakes, NJ, USA) according to the manufacturer's protocol. MDA-MB-468 cells were collected after transfection for 48 h, washed twice with cold PBS, and then suspended in a binding buffer containing Annexin V and PI. After incubation for 15 min at room temperature in the dark, the samples were immediately analyzed by using the FACSCalibur flow cytometer (BD Biosciences) and analyzed using FlowJo (FlowJo LLC, Ashland, OR, USA).

### Clinical data set analysis

To investigate the mRNA levels of TPD52, the public data sets EGAS00000000083, GSE19188, GSE12470 were analyzed in Oncomine (www.oncomine.org) according to the instructions. The online survival analysis software KM-plotter was employed for cancer survival analysis as previously described (Yu et al., 2017). The correlation analysis of TPD52 and Star-PAP was analyzed in R2 database.

### Statistical analysis

Data were presented as mean±standard deviation (s.d.) and analyzed for significance using GraphPad Prism 7 software (La Jolla, CA, USA). Two-tailed Student’s *t*-test was used to determine the statistical significance. *P<0.05, **P<0.01, ***P<0.001 were considered to be statistically significant.

## Supplementary Material

Supplementary information
