# Supplementary material for: Star-PAP regulates tumor protein D52 through modulating miR-449a/34a in breast cancer
Source: Biol Open. 2019 Nov 6;8(11):bio045914. doi: 10.1242/bio.045914 (PMC6899025; doi:10.1242/bio.045914)
Supplement: Supplementary information [file biolopen-8-045914-s1.pdf]

The demographic data of both sets of patient samples in Figure 5A and Figure S3

The dataset EGAS00000000083 collected 1992 breast samples and 144 controls (non-tumor samples) from tumor banks in UK and Canada. The tumor samples include breast cancer subtype (luminal A, luminal B, HER2 positive, normal-like or basal-like) (Curtis et al, Nature 486, 2012, 346-352). GSE19188 contained 91 tumor samples and 65 adjacent normal lung tissue samples from Netherlands (Hou et al, PLoS One 5, 2010, e10312). GSE12470 performed 43 ovarian cancer tissues comprising 8 early stage and 35 advanced stage tissues and 10 normal tissues from Japan (Yoshihara et al, Cancer Sci 100, 2009, 1421-1428).

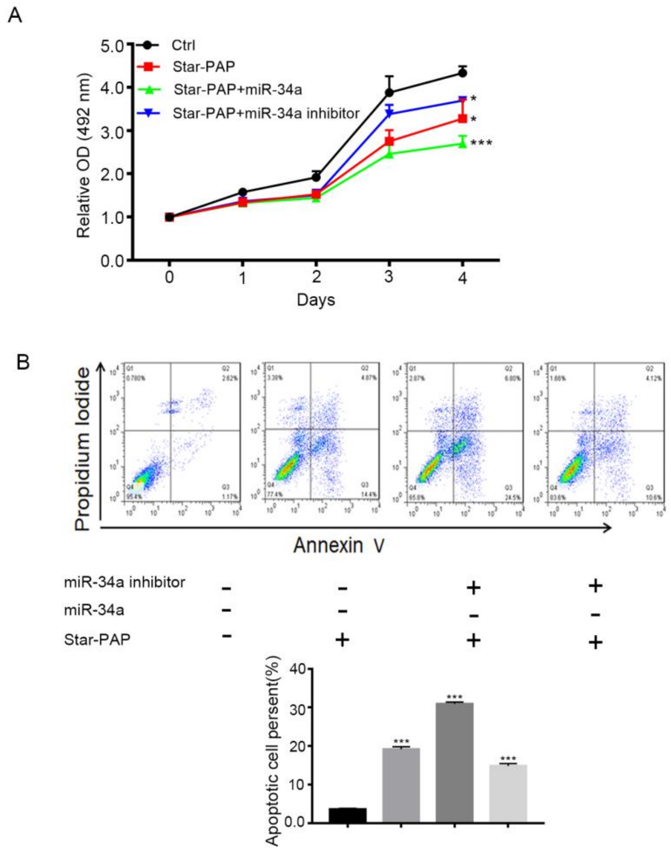

Figure S1. Star-PAP suppressed MDA-MB-468 proliferation and induced apoptosis through regulating miR-34a expression. A. Cells transfected with Star-PAP and miR-34a mimic or inhibitor were seeded in 4000/well, and cell viability was mesured by MTS. B. The same treatment with A for 48 h, and cell apoptosis was analysed. Data are means ± S.D (n=3) with three independent repeats, *p < 0.05, **p < 0.01, ***p < 0.001.

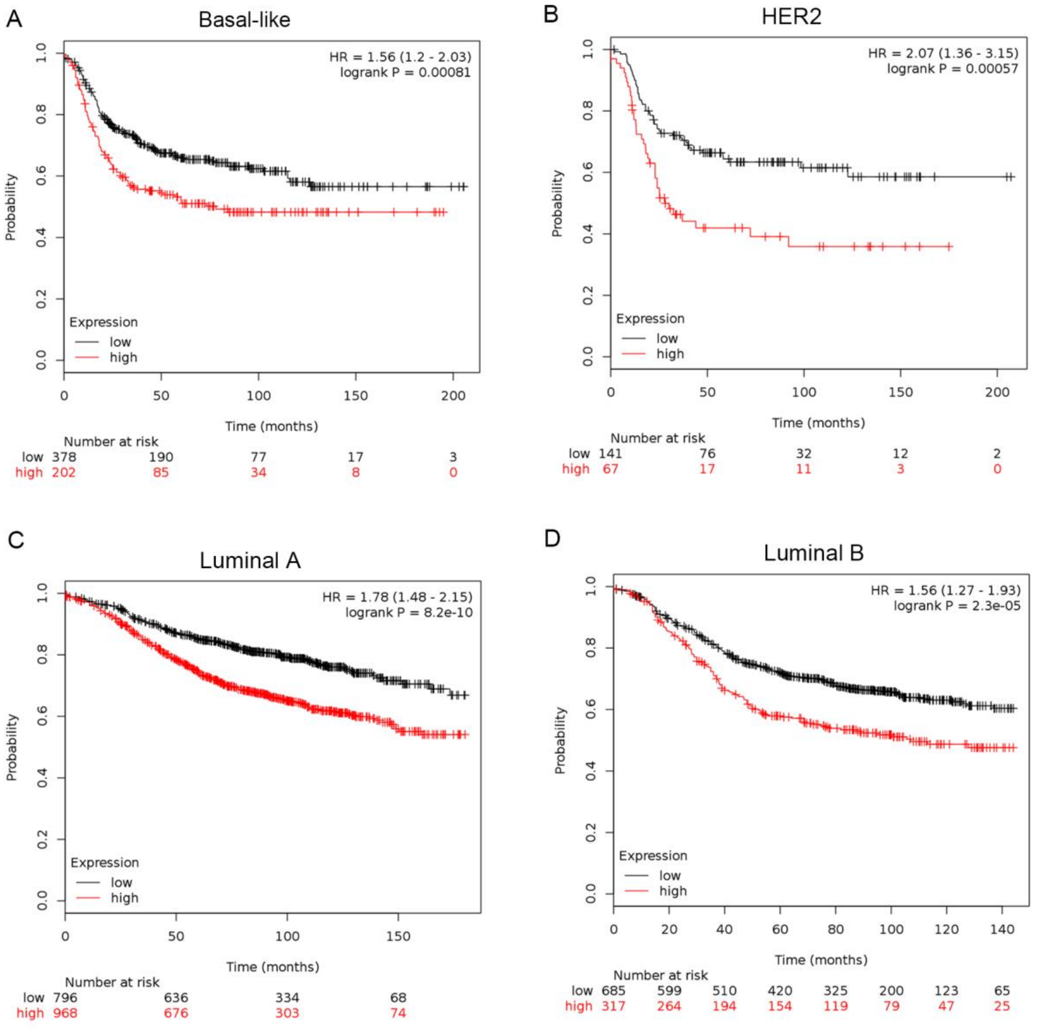

Figure S2. TPD52 correlates with prognosis of four substypes breast cancer patients. KM-plotter was exploited to investigate the correlation between TPD52 level and clinical prognosis. The relapse-free survival of breast cancer patients stratified by the expression level of TPD52 was shown. Four major breast cancer subtypes were investigated. (A) Basal-like; (B) HER2-enriched; (C) Luminal A; (D) Luminal B. Number of patients (n), log-rank p value and hazard ratio (HR) were shown.

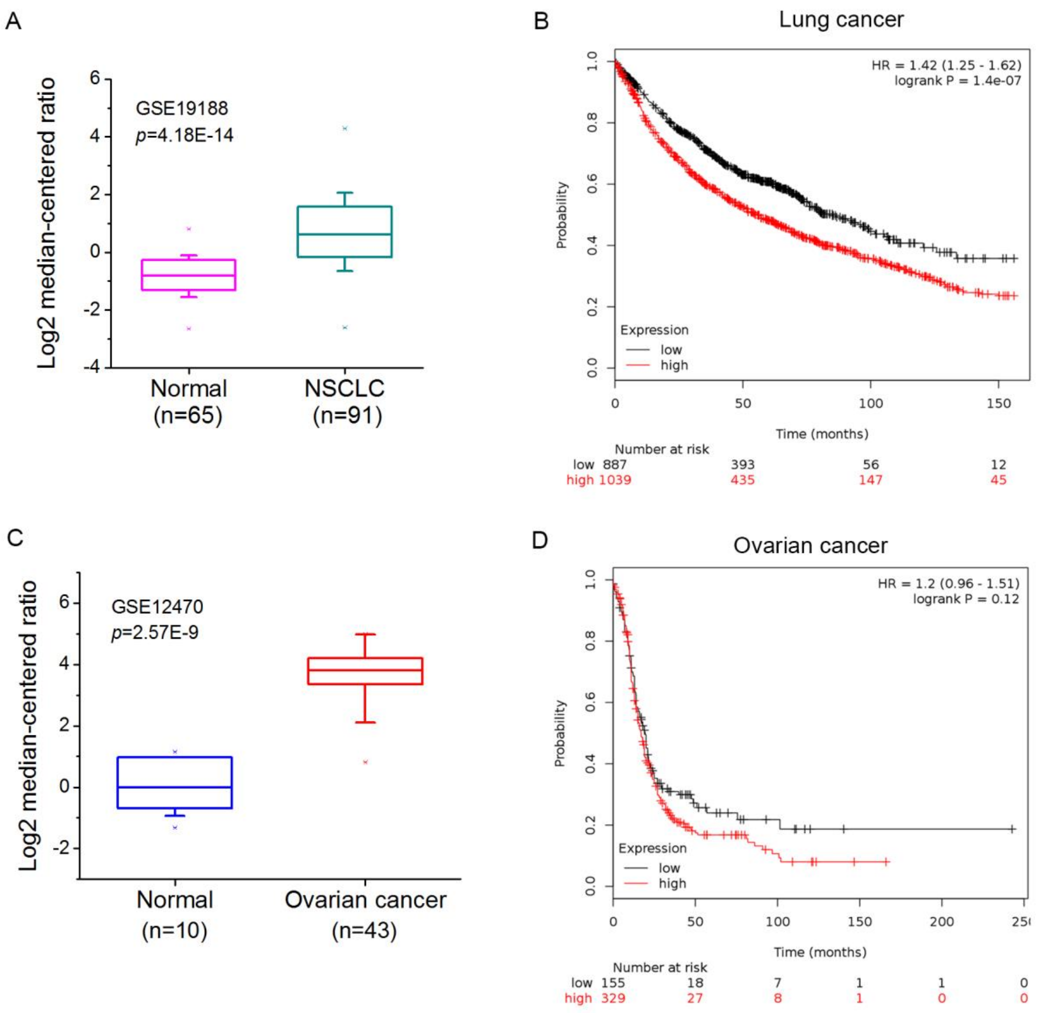

Figure S3. TPD52 correlates with prognosis of lung cancer and ovarian cancer patients. Box plots showed TPD52 expression levels in human lung cancer and ovarian cancer. KM-plotter was exploited to investigate the correlation between TPD52 level and clinical prognosis. (A) TPD52 expression levels in human lung cancer. Dataset accession number and p value were shown. (B) The relapse-free survival of breast cancer patients stratified by the expression level of TPD52. Number of patients, log-rank p value and hazard ratio (HR) were shown. (C) TPD52 expression level in human ovarian cancer. Dataset accession number and p value were shown. (D) The relapse-free survival of ovarian cancer patients stratified by the expression level of TPD52. Number of patients (n), log-rank p value and hazard ratio (HR) were shown.

Disease Models & Mechanisms • Supplementary information
